# 2D-DIGE based proteome analysis of wheat-*Thinopyrum intermedium* 7XL/7DS translocation line under drought stress

**DOI:** 10.1186/s12864-022-08599-1

**Published:** 2022-05-14

**Authors:** Fengkun Lu, Wenjing Duan, Yue Cui, Junwei Zhang, Dong Zhu, Ming Zhang, Yueming Yan

**Affiliations:** 1 Beijing Key Laboratory of Plant Gene Resources and Biotechnology for Carbon Reduction and Environmental Improvement, College of Life Science, Capital Normal University, Beijing, 100048 China; 2 College of Agricultural and Biological Engineering (College of Tree Peony), Heze University, 2269 Daxue Road, Heze, 274015 Shandong China

**Keywords:** Wheat, 7XL/7DS translocation, Grain proteome, 2D-DIGE, Drought tolerance

## Abstract

**Background:**

Drought stress is the most limiting factor for plant growth and crop production worldwide. As a major cereal crop, wheat is susceptible to drought. Thus, discovering and utilizing drought-tolerant gene resources from related species are highly important for improving wheat drought resistance. In this study, the drought tolerance of wheat Zhongmai 8601-*Thinopyrum intermedium* 7XL/7DS translocation line YW642 was estimated under drought stress, and then two-dimensional difference gel electrophoresis (2D-DIGE) based proteome analysis of the developing grains was performed to uncover the drought-resistant proteins.

**Results:**

The results showed that 7XL/7DS translocation possessed a better drought-tolerance compared to Zhongmai 8601. 2D-DIGE identified 146 differential accumulation protein (DAP) spots corresponding to 113 unique proteins during five grain developmental stages of YW642 under drought stress. Among them, 55 DAP spots corresponding to 48 unique proteins displayed an upregulated expression, which were mainly involved in stress/defense, energy metabolism, starch metabolism, protein metabolism/folding and transport. The *cis-acting* element analysis revealed that abundant stress-related elements were present in the promoter regions of the drought-responsive protein genes, which could play important roles in drought defense. RNA-seq and RT-qPCR analyses revealed that some regulated DAP genes also showed a high expression level in response to drought stress.

**Conclusions:**

Our results indicated that Wheat-*Th. intermedium* 7XL/7DS translocation line carried abundant drought-resistant proteins that had potential application values for wheat drought tolerance improvement.

**Supplementary Information:**

The online version contains supplementary material available at 10.1186/s12864-022-08599-1.

## Background

Wheat (*Triticum aestivum* L.) is one of the three main cereal crops widely cultivated in the world. Aside from being a main source of starch and energy, wheat also provides a number of ingredients that are vital or beneficial to health, particularly proteins, vitamins (especially B vitamins), dietary fiber and phytochemicals [1, 2]. However, along with the global climate change, the increased frequency of drought spells led to serious loss of crop yield [3–5].

To adapt to drought environment, plants have evolved a range of mechanisms at the morphological, physiological, biochemical, cellular, and molecular levels [6]. A key feature is that the phytohormone abscisic acid (ABA) is accumulated by hyperosmotic signal under drought stress, which in turn causes numerous adaptive responses in plants [7]. When exposed to drought, plants would produce reactive oxygen species (ROS), a sort of toxic byproducts of stress metabolism, which also acts primarily as signal transduction molecules regulating various pathways [8].

Stress-induced metabolic imbalance can result in activation of nicotinamide adenine dinucleotide phosphate (NADPH) oxidase, which can increase ROS production from other organelles such as mitochondria [9], peroxisomes [10] and chloroplasts [11]. ROS accumulation leads to oxidative signaling [12]. Meanwhile, the production of nitric oxide (NO) in plants, acting as a molecular messenger, is significant in the process of plant growth and defense responses such as signaling cascades responding to drought stress [13]. Generally, the diverse tolerance mechanisms of plant act in synchronization to survive in severe drought stress.

Proteins are involved in all intracellular events and play key roles in drought tolerance. Along with the rapid development of genomics, proteomics approach has become a powerful tool to uncover drought-tolerant proteins that have potential uses in crop improvement through marker-assisted selection [14]. To date, considerable proteomic work in crop plants has been performed to reveal the mechanisms of crop drought response in rice [15], wheat [16], maize [17], barley [18], soybean [11], bean [19] and sorghum [20]. In particular, wheat transcriptome and proteome analyses found that the developing grains contain abundant drought-resistant genes [21] and proteins that play important roles in response to drought stress during grain development and yield formation [22–26].

Wheat (2n = 6x = 42, AABBDD) is allohexaploid species with a giant genome up to 17 GB and has a large number of wild related species that served as an enormous gene pool for wheat genetic improvement. For instance, the genome-wide analysis showed that *Aegilops tauschii*, the D-genome progenitor of wheat, contained numerous genes for drought resistance [27]. *Triticum dicoccoides* is considered a potential drought-tolerance genes donor in wheat [28]. *Haynaldia villosa* (2n = 14, VV), a related species of wheat, carried abundant drought-responsive proteins [29]. As one of the wheat wild related species, *Thinopyrum intermedium* (2n = 6x = 42, E_1_E_1_E_2_E_2_XX, JJJ^S^J^S^StSt, E^e^E^e^E^b^E^b^StSt, or J^vs^J^vs^J^R^J^R^StSt) is a perennial forage crop distributed throughout the Mediterranean and Eastern Europe natively. *Th. intermedium* has high quantity of biomass and abundant gene resources for adversity resistance [30, 31], which was introduced into the United States for the first time in 1932 [30]. Gradually, *Th. intermedium* becomes a valuable resource used for enhancing disease resistance and yield potential of wheat via chromosomal translocation [32]. A single dominant gene resistant to stripe rust disease, designated as YrL693 and reported in 2014, was transferred from *Th. intermedium* into common wheat, which mapped to chromosome 1B [33]. The powdery mildew resistance gene PmL962, (a single dominant gene derived from *Th. intermedium* in the wheat line L962) was mapped to chromosome arm 2BS [34].

Different wheat stocks with *Th. intermedium* chromatin conferring resistance to barley yellow dwarf virus (BYDV) were developed through chromosome translocation and substitution. The common wheat Zhongmai 8601-*Th. intermedium* 7XL/7DS translocation line YW642 with BYDV resistance harbors distal segments of the long arm of *Th. intermedium* chromosome 7Ai-1 with the resistance gene Bdv2 [35]. Genome in situ hybridization (GISH) analysis showed that 10% of wheat chromosome 7DL distal or the whole 7DL were replaced by *Th. intermedium* chromatin in YW642 [36]. In the present study, we estimated the drought-tolerant performance of wheat Zhongmai 8601-*Th. intermedium* 7XL/7DS translocation line YW642, and used two-dimensional difference gel electrophoresis (2D-DIGE) based proteome approach to dissect drought-responsive proteins in the developing grains under drought stress. Our purpose is to discover the drought-tolerant proteins caused by 7XL/7DS translocation, which could provide potential gene resources for improving drought resistance of wheat cultivars.

## Results

### Changes of main agronomic traits of Zhongmai 8601 and YW642 under drought stress

**Fig. 1:**
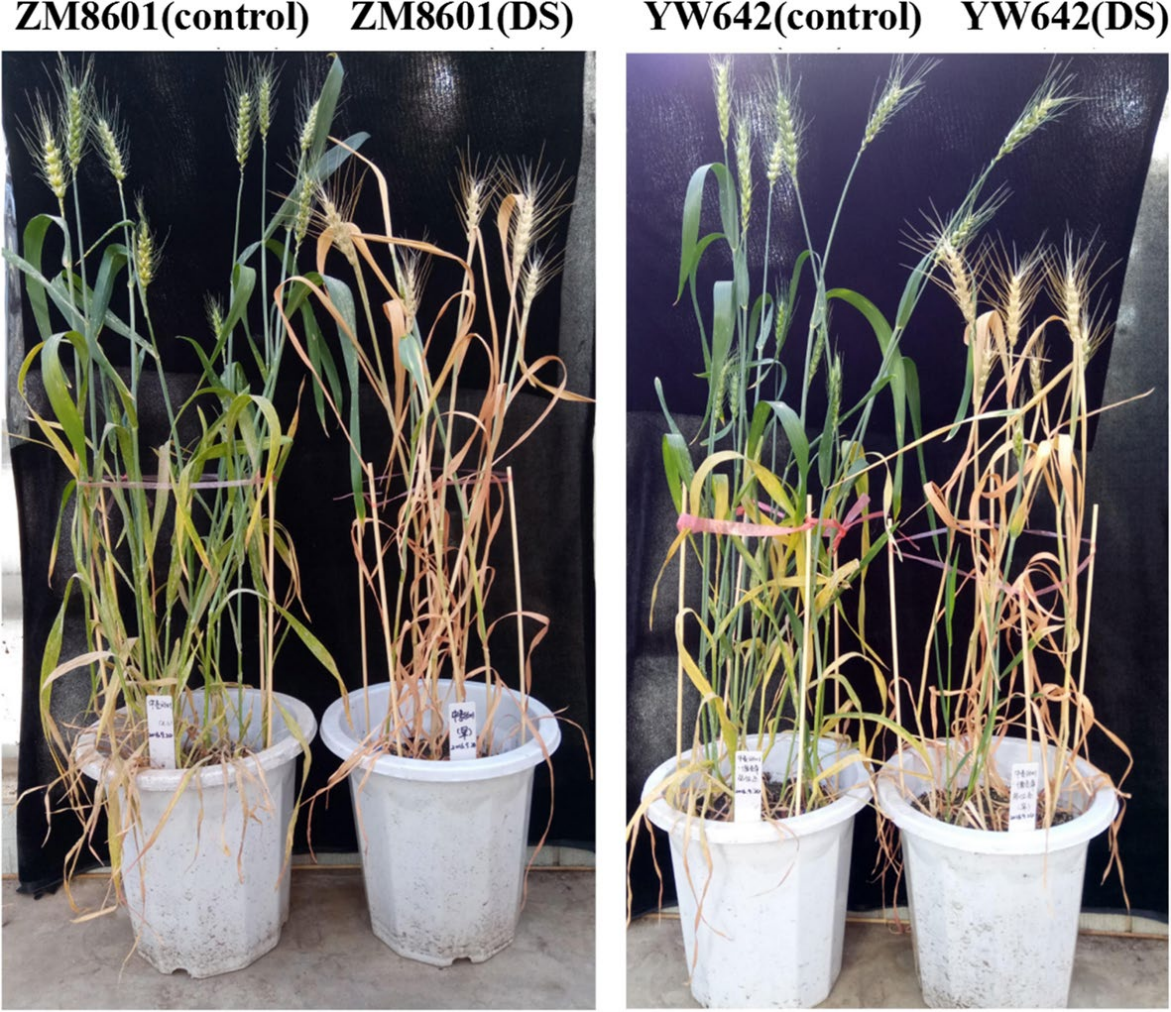
Performance of Zhongmai 8601 and wheat-*Thinopyron intermedium* 7XL/7DS translocation line (YW642) under drought stress

Analysis of main agronomic traits showed that 7XL/7DS translocation caused significant changes of plant growth and development under drought stress (Fig. 1), including significantly increase in plant height (13.24%), number of effective spikelets (45.96%), grain number per spike (68.33%) and 1000-grain weight (28.45%). On the contrary, number of tillers per plant and number of spikelets in YW642 were significantly decreased respectively by 17.41 and 7.06% compared to Zhongmai 8601. The ear length had no significant changes between Zhongmai 8601 and YW642 (Table 1). It should notice that unlike the performance under drought stress, many traits of YW642 had decreased under normal conditions, such as number of effective spikelets (− 5.95%), grain number per spike (− 21.65%). These results demonstrated that the 7XL/7DS translocation line had a better drought tolerance.

**Table 1:** Comparison of main agronomic traits between Zhongmai 8601 and Zhongmai 8601-*Th. intermedium* 7XL/7DS translocation line YW642 under drought stress

Materials	Plant height (cm)	Ear length (cm)	Number of tillers per plant	Number of effective spikelets	Number of spikelets	Grain number per spike	1000-grain weight (g)
Zhongmai 8601	71.13 ± 3.25	11.54 ± 0.43	2.12 ± 0.57	10.76 ± 0.78	17.62 ± 0.68	24.01 ± 1.32	41.15 ± 0.58
YW642	78.21 ± 3.43^b^	11.32 ± 0.41	1.66 ± 0.48^a^	10.12 ± 0.57^b^	16.12 ± 0.79^b^	18.81 ± 1.24^b^	42.98 ± 0.47^a^
Increased(+) /decreased (−) percentage	+ 9.95%	−1.91%	−21.69%	−5.95%	−8.51%	−21.65%	+ 4.45%
Zhongmai 8601 (DS)	56.73 ± 3.33	10.94 ± 0.69	2.24 ± 0.83	6.68 ± 2.29	17.27 ± 0.79	9.44 ± 4.40	26.43 ± 1.12
YW642 (DS)	64.24 ± 3.36^b^	11.14 ± 0.68	1.85 ± 0.75	9.75 ± 1.48^b^	16.05 ± 1.76^a^	15.89 ± 1.81^b^	33.95 ± 0.42^b^
Increased (+) /decreased (−) percentage	+ 13.24%	+ 1.83%	−17.41%	+ 45.96%	−7.06%	+ 68.33%	+ 28.45%

^a^ and ^b^ represent 5 and 1% significant differences, respectively

DS: Drought stress

### Grain proteome response of 7XL/7DS translocation line to drought stress

**Fig. 2:**
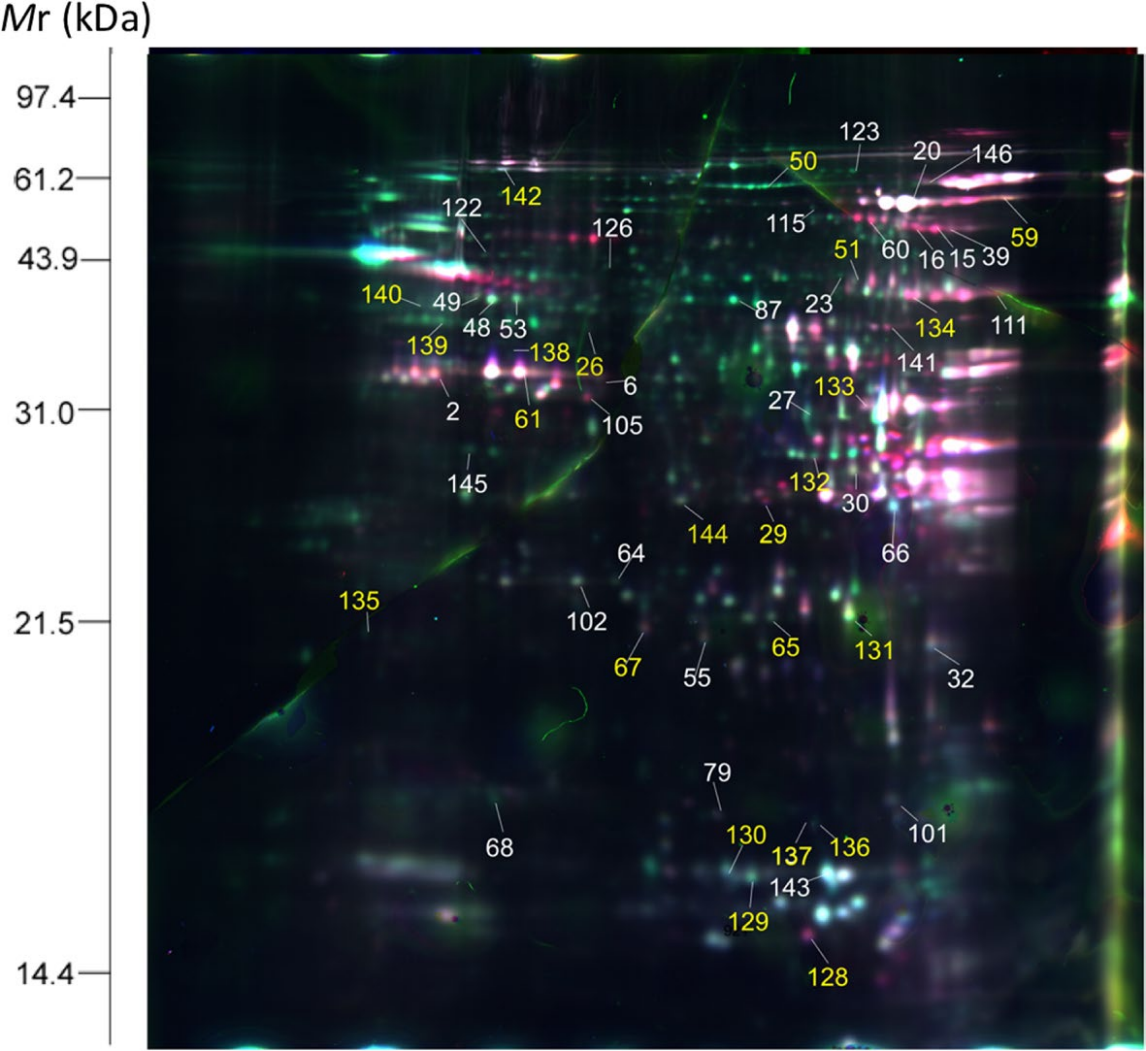
Identification of the upregulated drought-responsive differentially accumulated protein (DAP) spots in YW642 during drought stress. 2D-DIGE image exhibited 55 DAP spots during grain development at 20 days after flowering

**Fig. 3:**
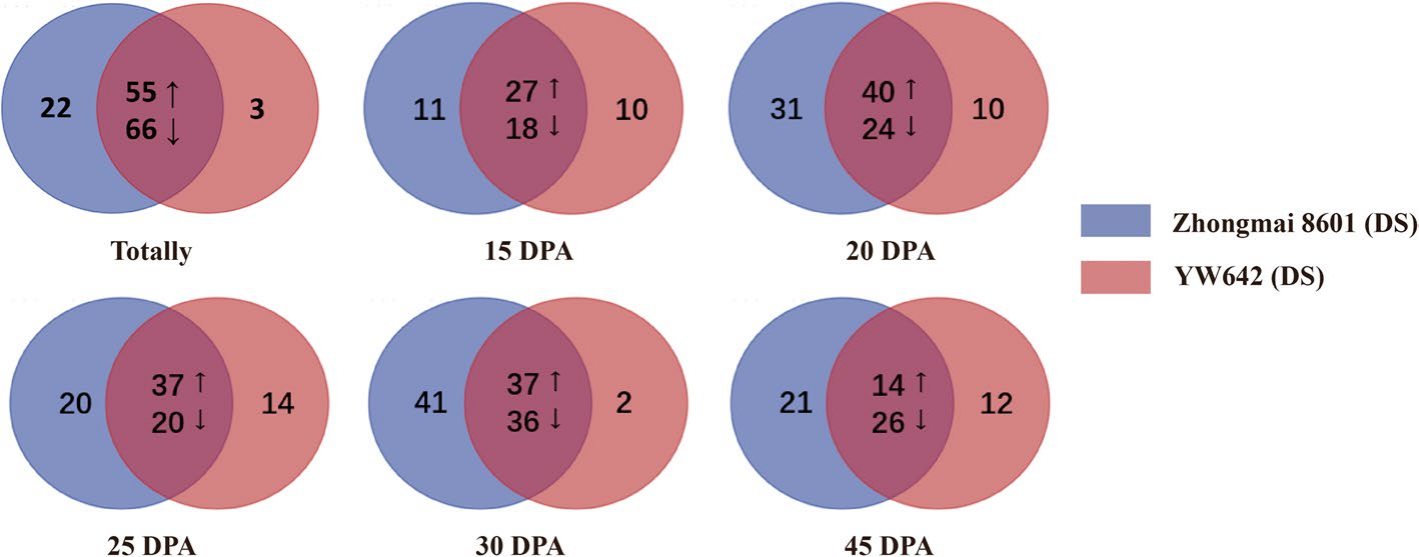
Number of differential accumulation protein (DAP) spots from 15, 20, 25, 30 and 45 DPA in wheat-*Thinopyrum intermedium* 7XL/7DS translocation line YW642 under drought stress. Five Venn diagrams represent the number of protein spots in the control and YW642 under drought stress group at five different grain developmental stages. The up and down arrows of the overlapping part represent the upregulated expression and downregulated expression in the corresponding period. Blue circle represents Zhongmai 8601 under drought stress while the red represents YW642 under drought stress

According to the previous report, 15-20 DPA were the key stages of wheat grain development, in which starch and storage proteins were rapidly synthesized and accumulated [21]. In this study, we used 2D-DIGE to identify DAPs at 20 DPA of grain development in the 7XL/7DS translocation line YW642 under drought stress (Fig. 2), and the detailed 2D-DIGE experimental designs were listed in Table S1. Subsequently, the dynamic expression profiling of DAPs at five grain developmental stages were detected by 2-DE (Fig. S1). In total, 146 DAP spots were identified. After collected and digested by trypsin, these DAP spots were successfully identified by MALDI-TOF/TOF-MS, which represented 113 unique proteins (Table S2). Compared to the control group, drought stress induced three specific DAP spots (spot 72, 73 and 121) and 121 common DAP spots with significant expression changes, including 55 upregulation (Table 2) and 66 downregulation. In particular, we found a DAP spot (spot 27) among the 55 upregulated proteins was identified as the same gamma-gliadin protein with spot 72. Likewise, two spots (spot 122 and spot 166) were found to represent the same pyruvate decarboxylase protein along with spot 121 (Table S2). We speculated that this may be caused by different types of protein post-translational modifications. Further analysis showed that 55 upregulated DAP spots in response to drought stress represented 48 unique proteins. The detailed information and peptide sequences of 55 upregulated drought-responsive DAPs were shown in Table S3. The dynamic changes of the 146 DAP spots during grain development showed that more and upregulated DAP spots mainly occurred at the middle stages (20-30 DPA) of grain filling (Fig. 3).

**Table 2:** The upregulated drought-responsive proteins in YW642 identified by MALDI-TOF/TOF-MS

Function classification/ spot ID	Accession No.	Protein Name (spot ID)	KOG number	Protein MW/PI	Protein Score C.I.%	Average %vol. ratio (YW642/control) at 15, 20, 25, 30, 45 DPA	*p*-value	Subcellular location prediction
**Stress/defense**								
spot 6	AAP80655	Formate dehydrogenase	KOG0069	28.99/8.61	100	2, 0.46, 4.42, 1.32, 8.06	0.012	Cyto
spot 29	KAF6988077	Glucose and ribitol dehydrogenase-like protein	KOG0725	31.88/6.54	100	1.88, 1.12, 1.89, 2.73, 0.36	0.021	Cyto
spot 30	KAF6988077	Glucose and ribitol dehydrogenase homolog	KOG0725	31.88/6.54	100	2.59, 1.36, 2.53, 2.07, 2.78	0.003	Cyto
spot 59	P93692	Serpin-Z2B	KOG2392	45.1/6.03	100	2.04, 1.49, 1.25, 4.19, 0.22	0.045	Chlo
spot 61	ACN59483	Serpin 1	KOG2392	43.1/5.44	100	1.6, 4.77, 2.13, 0.96, 3.82	0.024	Mito
spot 65	ADJ67792	Peroxidase 1	NA	39.26/8.14	100	2.09, 1.7, 3.2, 0.57, 0	0.021	Extr
spot 67	AAL71854	Dehydroascorbate reductase	KOG1422	23.46/5.88	100	2.13, 1.34, 3.13, 1.06, 0.46	0.034	Cyto
spot 68	CBM38934	Hsp26	KOG0710	27.42/5.82	100	2.12, 0.58, 0.11, 1.82, 0	0.001	Cyto
spot 105	ANW82830	Formate dehydrogenase, mitochondrial	KOG0069	41.64/6.51	100	1.91, 1.23, 0.47, 2.08, 2.07	0.015	Mito
spot 115	ABO70341	Pm3b-like disease resistance protein 15Q1	KOG4658	59.2/6.27	100	0.97, 1.1, 0.78, 2.79, 1.06	0.014	Cyto
spot 128	AAF02296	PR-4, partial	KOG4742	13.10/7.00	100	0, 1.1, 0.15, 3.97, 0.4	0.009	Wall
spot 135	BAA19099	2-Cys peroxiredoxin BAS1	KOG0852	23.31/5.71	100	0, 2, 3.87, 2.04, 0.98	0.013	Chlo^a^
spot 137	ACN59483	Serpin 1	KOG2392	43.10/5.44	100	2.06, 0.1, 0.41, 25.49, 0.63	0.012	Mito
spot 145	XP_044419905	Glyoxalase I	KOG2943	32.56/5.57	100	1.66, 3.02, 2.3, 2.04, 0	0.011	Cyto
**Energy metabolism**								
spot 23	XP_020185250	Dihydrolipoyl dehydrogenase 1, mitochondrial	KOG1335	58.81/7.63	100	2.65, 1.14, 2.14, 0, 0	0.019	Mito
spot 32	XP_044405132	Putative aconitate hydratase	KOG0452	93.9/5.66	100	5.24, 0.85, 8.77, 2.74, 0.94	0.026	Cyto
spot 102	CAC14917	Triosephosphate-isomerase	KOG1643	26.35/5.38	100	1.99, 2.2, 1.19, 0.28, 0.73	0.048	Cyto
spot 139	EMS51931	UTP--glucose-1-phosphate uridylyltransferase	KOG2638	50.86/5.76	100	2.28, 1.42, 1.81, 11.29, 0	0.036	Cyto
spot 140	CAA52636	ATP synthase beta subunit	KOG1350	59.21/5.56	100	2.08, 0.19, 2.27, 1.1, 1.45	0.017n	Mito
spot 141	AGH20062	enolase	KOG2670	48.10/5.49	100	1.18, 1.95, 2.06, 0.49, 0	0.034	Cyto
**Carbon metabolism**								
spot 15	CBH32516	Alpha-glucan phosphorylase, H isozyme,expressed	KOG2099	93.8 /7.60	100	0.25, 1.38, 1, 2.8, 0.29	0.019	Cyto
spot 16	AAM13694	Beta-D-glucan exohydrolase	NA	67.71/6.86	90	0.15, 2, 0.95, 2.59, 0.21	0.03	Chlo
spot 60	SPT18490	Endoglucanase	NA	35.3/8.71	100	0.03, 1.37, 1.24, 2.04, 0.3	0.034	Wall
**Starch metabolism**								
spot 143	AAY42618	Dimeric alpha-amylase inhibitor	NA	15.15/5.58	100	0, 0.86, 2.15, 4.22, 0	0.018	Cyto
**Nitrogen metabolism**								
spot 87	XP_037485906	Alanine aminotransferase 2	KOG0258	56.45/6.2	100	2.28, 2.03, 1.24, 0.18, 0	0.033	Cyto
spot 122	CBM36829	Pyruvate decarboxylase	KOG1184	65.83/5.43	100	2.71, 1.05, 2.59, 1.01, 0.71	0.016	Cyto
spot 126	CBM36829	Pyruvate decarboxylase	KOG1184	65.83/5.43	100	2.03, 2.08, 0.03, 0, 0	0.034	Cyto
**Protein metabolism/Folding**								
spot 48	AAL05264	Betaine-aldehyde dehydrogenase	KOG2450	54.4/5.44	100	1.94, 0, 2.35, 0.04, 0	0.028	Chlo
spot 131	AAU82107	20S proteasome beta 5 subunit	KOG0175	29.94/5.54	100	1.56, 0.2, 2.32, 1.04, 0.57	0.026	Cyto
spot 138	EMS58427	Elongation factor Tu	KOG0460	45.62/4.61	100	2.02, 1.36, 0.05, 0.48, 1.45	0.012	Chlo
spot 144	EMS61536	26S proteasome non-ATPase regulatory subunit 14	KOG1555	32.93/5.88	100	2.13, 2.7, 3.67, 0, 0	0.034	Cyto
**Transport**								
spot 101	ADK88900	Outer membrane channel protein OEP16-2	NA	17.95/6.7	95	0.03, 1.9, 1.63, 2.64, 1.39	0.045	Mito
spot 132	EMS55440	Kinesin-4	KOG0239	108.78/6.91	100	2.09, 0.74, 0.94, 2.05, 0.48	0.025	Cyto
**Storage protein**								
spot 26	ACB41346	Triticin	NA	62.76/6.43	100	2.55, 0.03, 1.49, 0, 0.03	0.048	Nucl
spot 27	AGO17690	Gamma-gliadin	NA	16.2/8.88	100	1.01, 2.19, 2.01, 1.95, 0	0.047	Vacu
spot 39	AFM30909	Globulin-3A	NA	66.3/8.48	100	42.79, 0.33, 2.03, 2.51, 0.36	0.037	Vacu
spot 50	EMS66832	Globulin-1 S allele	NA	56.9/9.1	100	3.33, 2.08, 1.11, 2.54, 0.13	0.036	Vacu
spot 51	EMS62417	Globulin-1 S allele	NA	55.59/7.77	100	0, 2.01, 3.58, 1, 0.95	0.047	Vacu
spot 53	ABS72144	Alpha gliadin	NA	33.47/8.19	100	0.19, 0.8, 2, 0.57, 5.42	0.033	Vacu
spot 64	AFM30909	Globulin-3A	NA	66.63/8.48	100	2.28, 0.8, 1.4, 0.47, 0.04	0.038	Vacu
spot 79	ACJ65513	Globulin 3C	NA	38.41/9.15	100	1.39, 0.07, 2.14, 2.15, 2	0.012	Vacu
spot 111	AFM30909	Globulin-3A	NA	66.63/8.48	100	1.19, 5.85, 0, 1.14, 2.12	0.028	Vacu
spot 129	ACJ65515	Globulin 3B	NA	56.90/7.36	100	1.34, 0.13, 3.55, 1.41, 0.48	0.023	Vacu
spot 130	ACJ65514	Globulin 3	NA	66.31/7.78	100	0, 0.17, 3.79, 1.66, 3.7	0.047	Vacu
spot 133	EMS60011	12S seed storage globulin 1	NA	63.83/6.62	100	0.55, 1.04, 2.02, 3.25, 5.56	0.028	Vacu
spot 134	EMS62417	Globulin-1 S allele	NA	55.30/7.77	100	3.5, 1.06, 1.18, 2.07, 0.77	0.037	Vacu
spot 136	AAB27108	Triticin precursor	NA	56.92/9.37	100	1.86, 2.86, 2.2, 3.57, 0.85	0.039	Vacu
spot 142	AKW50839	High molecular weight glutenin subunit	NA	90.51/6.15	100	14.55, 0.4, 6.07, 1.38, 1.86	0.018	Nucl
spot 146	ACJ65514	Globulin 3	NA	66.31/7.78	100	0.88, 0, 2.23, 4.7, 0	0.019	Vacu
**Others**								
spot 2	KAF6997295	BJ297754 Y	NA	38.5/5.02	100	0, 2.43, 0.34, 1.82, 4.52	0.03	Chlo
spot 20	KAF6997295	G356.108B02F010919	NA	36.4/5.13	100	2.74, 2.03, 1.75, 1.07, 0.95	0.034	Nucl
spot 49	EMS54324	Hypothetical protein TRIUR3_03549	KOG0710	16.82/6.19	100	0.62, 1.2, 0.11, 2.17, 0.98	0.021	Nucl
spot 55	EMS66582	Hypothetical protein TRIUR3_24891	NA	22.69/8.42	100	0.21, 0.37, 1.4, 1.27, 2.01	0.034	Nucl
spot 66	KAF7049137	G608.111J02F010910	NA		100	0.18, 1.14, 2.95, 0.58, 0.4	0.001	Nucl
spot 123	KAF6997295	Hypothetical protein F775_14176	NA	45.64/7	100	1.05, 5.69, 4.4, 2.22, 1.31	0.018	Nucl

^a^The verified subcellular localization is marked with an asterisk. And the ratio greater than 1.5 means upregulated

**Fig. 4:**
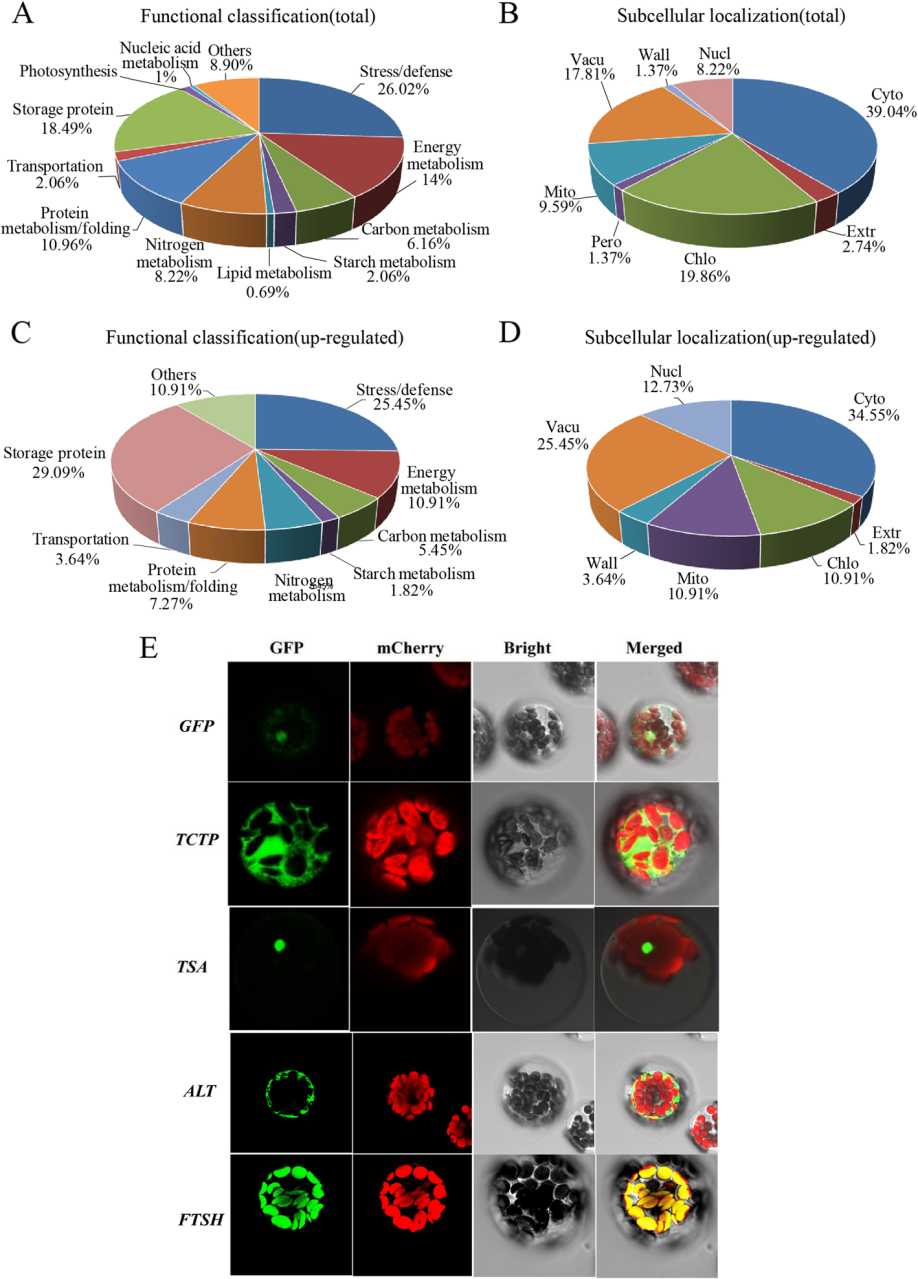
Functional classification and subcellular location of the 55 upregulated drought-responsive DAPs identified in the 7XL/7DS translocation line YW642. **A** Function classification of the 146 drought-responsive proteins in YW642 under drought stress. **B** Subcellular localization prediction of the 146 drought-responsive proteins in YW642 under drought stress. **C** Function classification of the 55 upregulated drought-responsive proteins in YW642 under drought stress. **D** Subcellular localization prediction of the 55 upregulated drought-responsive proteins in YW642 under drought stress. **E** Subcellular localization assay via Arabidopsis protoplast cells. GFP: GFP fluorescence signal. Green fluorescence indicates the location of DAPs; mCherry: red fluorescent dye; Red fluorescent signal indicates the location of chloroplasts in protoplasts; Bright light: field of bright light; Merged: emergence of the GFP fluorescence signal, mChery red fluorescent signal and bright light field; Control: 16318-35S-GFP empty vector. Scale bar = 10 μm

The functional classification of 146 DAP spots included 12 functional categories: 26.02% in stress/defense, 18.49% in storage proteins, 14.38% in energy metabolism, 10.96% in protein metabolism/folding, 8.22% in nitrogen metabolism, 6.16% in carbon metabolism, 2.06% in starch metabolism, 2.06% in transportation, 1.37% in photosynthesis, 0.69% in lipid metabolism, 0.69% in nucleic acid metabolism, and 8.9% in others (Fig. 4A). The website prediction results of subcellular localization in Fig. 4B showed that these DAPs were mainly located in cytoplasm (39.04%), chloroplast (19.86%), vacuole (17.81%), mitochondria (9.59%), nucleus (8.22%), extracellular (2.74%), peroxisome (1.37%) and cell wall (1.37%).

The 55 upregulated DAP spots identified in the 7XL/7DS translocation line under drought stress could be divided into nine functional categories: 16 in storage proteins, 14 in stress/defense, 6 in energy metabolism and others, 4 in protein metabolism/folding, 3 in nitrogen metabolism and carbon metabolism, 2 in transportation and one in starch metabolism (Fig. 4C). The subcellular localization results of these upregulated DAP spots by website prediction included 19 in cytoplasm, 14 in vacuole, 7 in nucleus, 6 in chloroplast, 6 in mitochondria, 2 in wall and one in extracellular (Fig. 4D).

Four representative proteins related to drought response were selected for further subcellular localization verification in Arabidopsis protoplasts, including translationally controlled tumor protein (TCTP, spot 10), 2-Cys peroxiredoxin BAS1 (TSA, spot 135), alanine aminotransferase 2 (ALT, spot 109), and ATP-dependent zinc metalloproteinase (FTSH2, spot 12). Compared to the control 3449 empty vector, their subcellular localization could be determined: TCTP and ALT in the cytoplasm, TSA in the nucleus, and FTSH2 in the chloroplast (Fig. 4E).

### Dynamic accumulation profiling of the upregulated drought-responsive DAPs identified in the 7XL/7DS translocation line

**Fig. 5:**
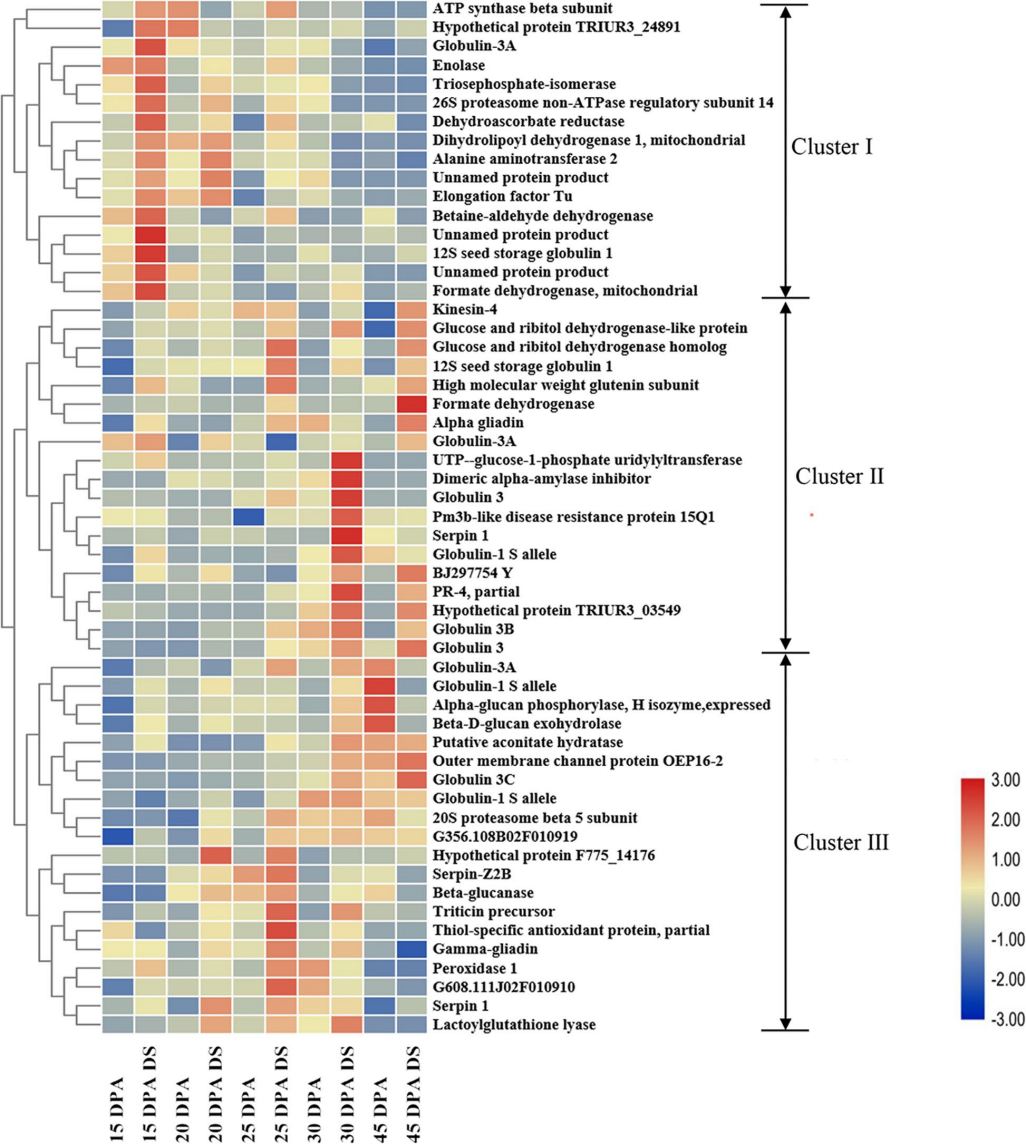
Dynamic accumulation profiling of the 55 upregulated drought-responsive DAPs identified in the 7XL/7DS translocation line YW642. Cluster analysis of the 55 DAPs identified in wheat-*Thinopyrum intermedium* 7XL/7DS translocation line during different grain development stages under drought stress. Each column stands for samples from different treatments and five grain development stages (15, 20, 25, 30, 45 days post anthesis, DPA). Each row uses a color ratio based on the relative ratio to display the changes in DAP spot, blue means low expression, red means high expression and yellow means the middle

Heat map cluster analysis showed the dynamic accumulation profiling of 55 upregulated DAP spots during grain development in the 7XL/7DS translocation line (Fig. 5). In general, 55 DAP spots were categorized into three clusters (I-III). Compared with the control group, cluster I with 16 DAP spots (15 proteins) showed a significant upregulation from early to middle grain filling stages, and these proteins mainly participated in energy metabolism, protein metabolism/folding, stress/defense, and nitrogen metabolism. Cluster II containing 19 DAP spots (17 proteins) displayed an upregulated trend in the late grain filling stages, which mainly involved in stress/defense, transportation, storage protein and starch metabolism. Cluster III included 20 DAP spots (20 proteins) and exhibited a significantly upregulated expression at the mid-late grain filling stages. These proteins mainly involved in storage, stress/defense, carbon metabolism, protein metabolism, transportation, and energy metabolism.

Since different types of protein post-translational modifications generally occurred during grain development [37], protein spots 39, 64 and 111 with different molecular mass and isoelectric points (Table S2) were identified as the same globulin-3A protein, but they were highly expressed at different periods: spot 39 and 64 highly expressed in 15 DPA and spot 111 with a higher expression level in 20 DPA. Similarly, serpin 1 included spots 61 and 137 and globlin-1 S allele contained spots 50, 51 and 134. In general, the upregulated drought-responsive DAP spots identified in the 7XL/7DS translocation line at early-middle filling stages were mainly involved in energy metabolism, protein metabolism/folding, stress-defense and nitrogen metabolism, while those at middle-late filling stages mainly participated in stress defense and storage substance synthesis.

### Interaction network analysis of the upregulated drought-responsive DAPs

**Fig. 6:**
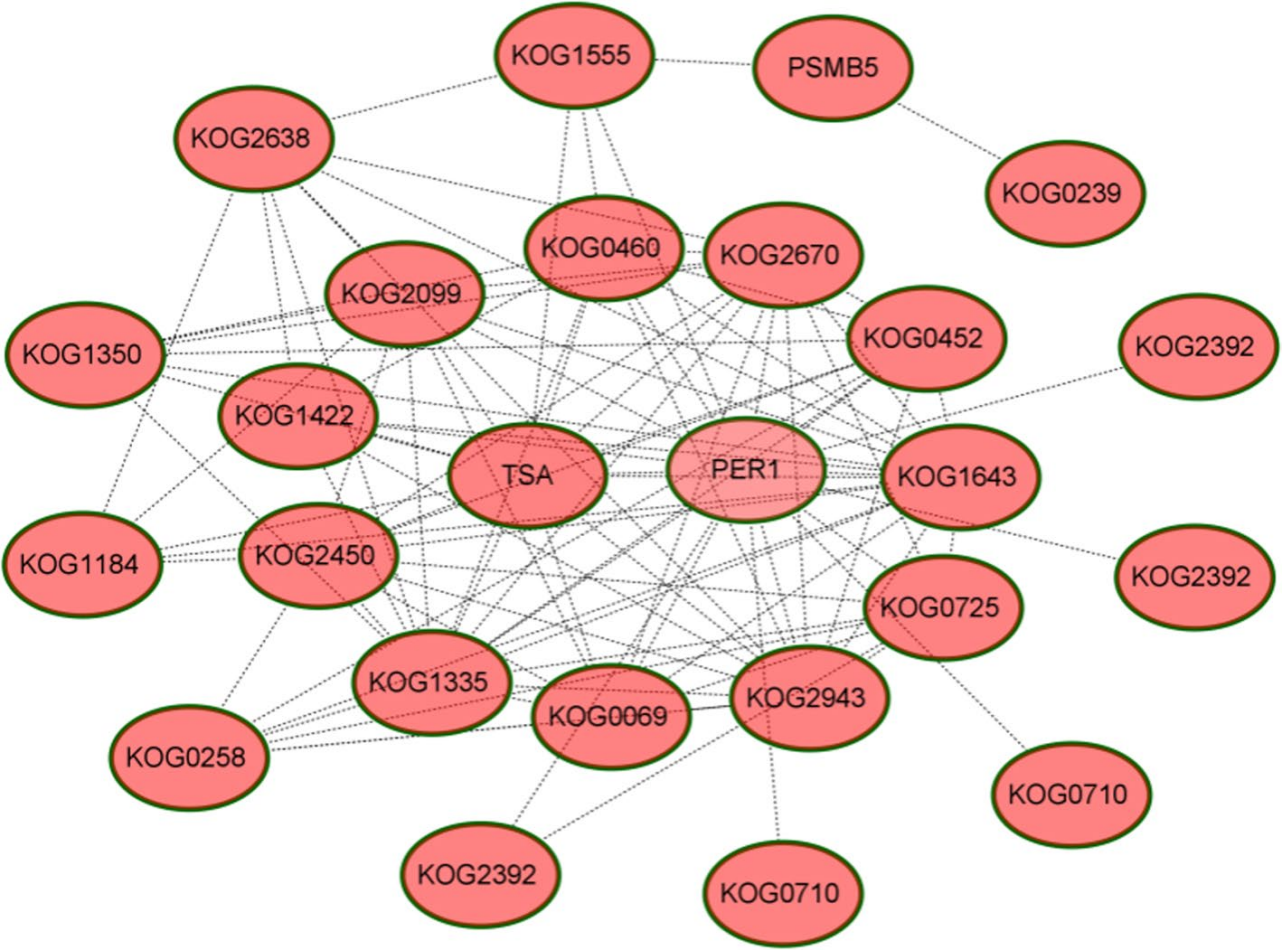
Protein-protein interaction (PPI) networks of the 25 drought-responsive DAPs identified in the 7XL/7DS translocation line YW642. KOG2099: Alpha-1,4 glucan phosphorylase (spot 15); KOG1335: Dihydrolipoyl dehydrogenase (spot 23); KOG0710: SHSP domain-containing protein (spot 49); KOG2392: Serpin-Z2B (spot 59); PER1: Peroxidase 1 (spot 65); KOG0258: Aminotran_1_2 domain-containing protein (spot 87); KOG1643: Triosephosphate isomerase (spot 102); KOG0069: Formate dehydrogenase (spot 105); KOG1184: Pyruvate decarboxylase (spot 122); KOG4742: PR4B (spot 128); KOG0175: Proteasome subunit beta (spot 131); TSA: 2-Cys peroxiredoxin BAS1 (spot 135); KOG0460: Elongation factor Tu (spot 138); KOG2638: UTP-glucose-1-phosphate uridylyltransferase (spot 139); KOG1350: ATP synthase beta subunit (spot 140); KOG2943: Glyoxalase I (spot 145)

In order to reveal the interaction and relationship between different proteins, protein-protein interaction (PPI) networks of the upregulated DAPs under drought stress were generated using the STRING (Fig. 6). This network consisted of 25 proteins (PPI enrichment *p*-value: < 1.0e-16), which were mainly involved in stress/defense, energy metabolism and storage. Among them, two proteins caused by the 7XL/7DS translocation occupied a relatively important position: peroxidase I (PER1, spot 65) and 2-Cys peroxiredoxin BAS1 (TSA, spot 135), which showed a close interaction with other proteins. As a member of peroxidases, they can remove active oxygen in plants, indicating that peroxidase may play an important role in coping with drought stress.

### Analysis of *cis-acting* elements in the promoter region of the drought-responsive protein genes in the 7XL/7DS translocation line

Every gene contains a particular combination of *cis-acting* regulatory sequence elements in the upstream promoter regions, which determines its temporal and spatial expression [38]. The *cis-acting* regulatory elements are crucial transcriptional gene regulatory units, which controls numerous biological events and stress responses. The compositions of main *cis-acting* elements related to drought response in the promoter regions of the upregulated protein genes under drought stress were identified (Table 3). The results showed that the *cis-acting* elements involved in hormone and environmental stress response were particularly abundant. Among phytohormone responsive elements, abscisic acid responsiveness *cis-acting* element ABRE was the most abundant, following by MeJA (methyl jasmonate)-responsiveness (TGACG-motifs and CGTCA-motifs), gibberellin-responsiveness (GARE-motif and TATC-box), auxin-responsive element (TGA-elements and AuxRR-core) and ethylene-responsiveness (ERE). The *cis*-regulatory elements involved in environmental stress response mainly included ARE (regulatory element essential for the anaerobic induction), GC-motif (enhancer-like element involved in anoxic specific inducibility), TC-rich repeats (*cis-acting* element involved in defense and stress responsiveness), and MBS (MYB binding site involved in drought-inducibility). It is known that the transgenic expression of MYB transcription factor can increase drought tolerance [39]. Thus, the rich *cis*-elements of MBS present in the 7XL/7DS translocation line could play important roles in drought tolerance. The *cis*-elements analysis also showed the 7XL/7DS translocation line could respond other abiotic stresses such as low temperature (LTR) and wound (WUN-motif). These *cis-acting* elements lay the structural foundation for gene expression in response to adverse environments.

**Table 3:** The *cis-acting* elements of the upregulated drought-responsive DAP genes caused by 7XL/7DS translocation

Spot ID	Protein name	Hormone responsive elements								Environmental stress-related elements					
		GARE-motif	TGA-element	TATC-box	AuxRR-core	ERE	TGACG-motif	CGTCA-motif	ABRE	LTR	WUN-motif	GC-motif	ARE	TC-rich repeats	MBS
6	Formate dehydrogenase	2	1	0	0	0	2	2	7	2	1	4	2	0	0
29/30	Glucose and ribitol dehydrogenase-like protein	0	0	0	1	0	5	5	10	0	0	2	0	0	0
61/137	serpin 1	2	0	1	1	0	2	2	1	1	0	0	2	1	1
65	Peroxidase 1	2	1	2	0	2	2	2	4	0	0	0	0	0	1
67	Dehydroascorbate reductase	0	1	0	0	0	1	1	3	0	1	0	0	1	0
128	PR-4, partial	0	2	0	0	0	3	3	7	1	0	2	0	0	3
135	Thiol-specific antioxidant protein, partial	1	1	0	2	1	1	1	3	0	0	0	2	0	2
59	Serpin-Z2B	1	0	0	0	0	0	0	3	1	2	0	3	3	3
105	Formate dehydrogenase, mitochondrial	0	0	0	0	0	1	1	6	2	1	1	3	0	0
115	Pm3b-like disease resistance protein 15Q1	2	0	0	0	0	1	1	3	1	0	0	1	0	1
48	betaine-aldehyde dehydrogenase	1	3	0	1	0	2	2	5	0	0	0	1	0	1
131	20S proteasome beta 5 subunit	2	0	0	0	0	2	2	6	0	0	0	1	3	1
138	Elongation factor Tu	0	0	0	0	0	3	3	3	1	0	0	2	0	1
144	26S proteasome non-ATPase regulatory subunit 14	1	1	0	0	0	4	4	5	0	0	0	6	0	1
145	Glyoxalase I	0	0	0	0	0	3	3	6	0	1	1	0	0	1

### RNA-seq expression analysis of the drought-responsive DAP genes

**Fig. 7:**
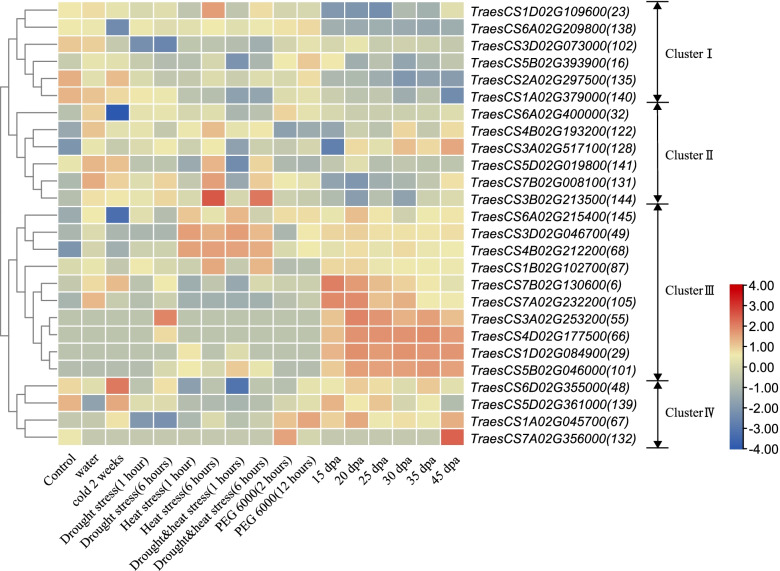
RNA-seq expression profiles of the 26 drought-responsive DAP genes in the 7XL/7DS translocation line YW642 under various biotic and abiotic stresses. The number in bracket is the DAP spot number

The public transcriptome database of wheat was used to analyze the transcription expression patterns of 55 DAP genes during grain development in response to different stressors and data of 26 genes were obtained from it (Fig. 7). These DAPs from 7XL/7DS translocation line showed significantly upregulated expression under drought stress (Fig. 5). In total, 26 DAP genes could be divided into four clusters. In detail, a total of 14 genes in Cluster III (10 genes) and Cluster IV (4 genes) had high expression levels during grain development periods. Among them, 4 genes could respond to drought stress, 5 genes could respond to drought & heat stress. Comparatively, the expression levels of Cluster I (5 genes) and Cluster II (6 genes) during grain development periods are low, with only 2 genes were highly expressed in the later stages, but there were 5 genes showed higher expression levels under drought stress.

### Dynamic transcriptional expression profiling of the drought-responsive DAP genes during grain development by RT-qPCR

**Fig. 8:**
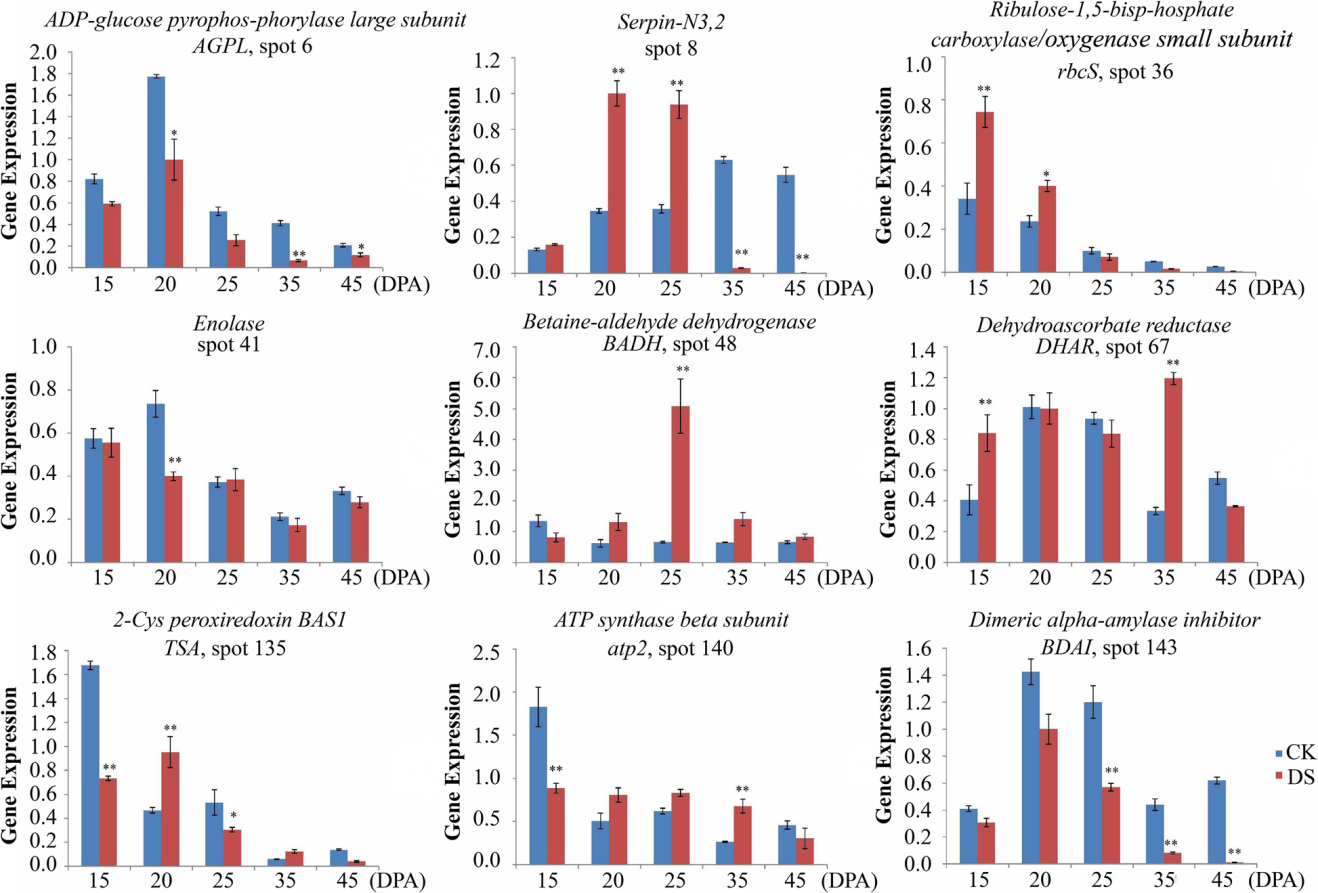
RT-qPCR analysis of nine representative DAP genes from 7XL/7DS translocation line YW642 during grain development in response to drought stress. *AGPL*, ADP-glucose pyrophos-phorylase large subunit; *enolase*, enolase coding gene; *TSA*, 2-Cys peroxiredoxin BAS1; *BADH*, betaine-aldehyde dehydrogenase; *atp2*, ATP synthase beta subunit; *rbcS*, ribulose-1,5-bisp-hosphate carboxylase/oxygenase small subunit; *DHAR*, dehydroascorbate reductase; *BDAI*, Dimeric alpha-amylase inhibitor. Statistically significant differences are calculated based on an independent Student’s t-tests: *p < 0.05; **p < 0.01

Nine representative drought-responsive DAP genes were selected for further transcriptional expression analysis, and their dynamic expression profiles were detected by RT-qPCR (Fig. 8). These genes included ADP-glucose pyrophos-phorylase large subunit (*AGPL*, spot 5), serpin-N3.2 (spot 8), ribulose-1,5-bisphosphate carboxylase/oxygenase small subunit (*rbcS*, spot 36), enolase (spot 41), betaine-aldehyde dehydrogenase (*BADH*, spot 48), dehydroascorbate reductase (*DHAR*, spot 67), 2-Cys peroxiredoxin BAS1 (*TSA*, spot 135), ATP synthase beta subunit (*atp2*, spot 140), and dimeric alpha-amylase inhibitor (*BDAI*, spot 143). The gene-specific primers designed by using online Primer3Plus are listed in Table S4. Compared with the control, six DAP genes (*BADH*, *DHAR*, *TSA*, *atp2*, *rbcS* and *serpin-N3.2*) generally showed an upregulated expression during grain development in response to drought stress, particularly at the middle filling stages. The remaining three DAP genes (*AGPL*, *enolase* and *BDAI*) displayed a clear downregulated expression trend. Among them, *BADH*, *DHAR*, *TSA* and *atp2* showed a similar expression trend in both transcription and translation level, but the remaining five DAP genes had a poor consistency.

## Discussion

ROS serves as an important signal transduction molecule, short-lived and highly reactive [40]. Abiotic stress could activate the formation of ROS that plays a dual role in plant response to abiotic stress [41]. In higher plants, high concentration of ROS species will destroy the main cell components of the organism, while moderate concentration of ROS species can act as a regulating medium to protect cells and rebuild redox homeostasis [42]. Numerous peroxidases, reductases, and dehydrogenases are involved in ROS processing [43]. For instance, ascorbate peroxidase (APX) serves as an indispensable antioxidant enzyme that can keep ROS levels and maintain the cellular homeostasis under stress [44]. As a kind of major antioxidants, peroxidases play a crucial role in ROS detoxification under adverse conditions [40]. Based on the subcellular location and glycoprotein nature, peroxidases contain two categories in plants: class I and class III. Peroxidase 1 belongs to the latter, which uses electrons from various donor molecules to catalyze the reduction of H_2_O_2_ [45], and then were transferred into the cell wall or surrounding medium and vacuoles [46]. For example, the member of peroxidase superfamily AtPrx12 (At1g71695) could response to oxidative stress and involve in oxidation reduction in Arabidopsis [47]. In this study, we found that peroxidase 1 (spot 65) in wheat-*Th. intermedium* 7XL/7DS translocation line was upregulated under drought stress (Fig. 2, Table 2), which could enhance plant drought tolerance via clean up excessive ROS. 2-Cys peroxiredoxin was a large family, widely distributed in all organisms. They can catalyze the transfer of electrons from sulfhydryl residues to peroxides, reducing alkyl hydroperoxides and hydrogen peroxide and other substances [48]. The expression of 2-Cys peroxiredoxin BAS1 was upregulated in drought-tolerant wheat varieties, indicating that it might play an important role in drought resistance [49]. The study of the reaction mechanism of plant 2-Cys peroxiredoxin showed that it had broad substrate specificity and could even reduce complex lipid peroxides. Thus, 2-Cys peroxiredoxin not only has the function of peroxide detoxification, but also might function as a structural redox sensor in chloroplasts [50]. The 7XL/7DS translocation caused the upregulation of 2-Cys peroxiredoxin BAS1 protein (Thiol-specific antioxidant protein, TSA, spot 135) under drought stress (Fig. 5), which could contribute to improve drought tolerance.

Dehydroascorbate reductase (DHAR, spot 67), one of the key enzymes related to oxidative stress, played a crucial role in maintaining the normal level of ascorbic acid (AsA, vitamin C) by circulating oxidized ascorbic acid [51]. As previously reported, AsA is a very potent antioxidant that is synthesized in plant mitochondria and then transported to other cell regions to eliminate ROS [52, 53]. Monodehydroascorbate reductase (MDHAR) and dehydroascorbate reductase are indispensable for the regeneration of AsA to maintain the ability of ROS scavenging. Simultaneous expression of the two genes in *Brassica rapa* produced a synergistic effect that was more effective than the overexpression of a single gene, which increased the tolerance of stress more effectively even in the severe condition [54]. We found that DHAR, one of the two vital enzymes functioning in the reproduction of AsA, was upregulated in the 7XL/7DS translocation line in response to drought stress (Fig. 2, Table 2). DHAR has showed to function in ozone tolerance [55], salt tolerance [56], and aluminum resistance [57] together with drought tolerance [58]. In Arabidopsis, *Nicotiana tabacum* and *Triticum aestivum*, DHAR was not only found in the cytoplasm, but also in the chloroplast, and its overexpression enhanced the ability of plants to resist salt and cold stresses [59].

Under various abiotic stresses, plants produce a high amount of methylglyoxal (MG), an α-oxoaldehyde compound which is highly reactive cytotoxic can form advanced glycation end products and deactivate the antioxidant system [60]. It is primarily as a byproduct of several metabolic pathways such as glycolysis, lipid peroxidation and others [61]. In the PPI networks, we found the existence of an interaction network mainly involving three proteins, namely glyoxalase I (KOG2943, spot 145), triosephosphate-isomerase (KOG1643, spot 102) and pyruvate decarboxylase (KOG1184, spots 121, and 126) (Fig. 6). Glyoxalase I is an important component of the glyoxalase system, which could remove MG, thereby improving the ability of plants to cope with abiotic stress [62]. We speculate that under drought conditions, the upregulation of triosephosphate-isomerase and pyruvate decarboxylase [63] is the result of high glycolytic flux, and at the same time, plants activate the methylglyoxal system to remove excess MG.

Protease inhibitors are typically expressed constitutively in many plants, especially after induction of biotic stress and in storage tissues such as seeds and tubers [64]. Serpins are a kind of protease inhibitors that are ubiquitous in plant kingdom, which constitute a superfamily of multifunctional proteins involved in the regulation of complex proteolytic systems [65]. Serpin acted as one of drought related proteins identified in CS-1S^l^(1B) substitution line [26]. Proteomic analysis also revealed that serpins served as drought stress-related proteins and could play a role in the regulation of plant defense [24, 66, 67]. We found that wheat-*Th. intermedium* 7XL/7DS translocation caused the upregulation of three serpin spots (59, 61 and 137) under drought stress (Fig. 5, Table 2). Ubiquitin-proteasome system (UPS) is involved in regulating almost every aspect of plant growth and development [68, 69]. UPS could precisely regulate plant hormone signaling by affecting protein activity, localization, assembly, and ability of interaction. Abscisic acid (ABA) is a major plant hormone involved in all the stages of plant growth, and the process of UPS from ABA perception to function was elaborated [70]. The 26S proteasome complex is the main molecular machine responsible for protein degradation in eukaryotic cells [71], and 26S proteasome containing multiple proteins took a prominent role in plant abiotic stress signaling [72]. In this study, an upregulated drought responsive protein 26S proteasome non-ATPase regulatory subunit 14 (spot 144) was identified in the 7XL/7DS translocation line, which also is a member of the 26S proteasome and may be related to the drought stress response [73, 74]. In addition, the 20S proteasome, as the catalytic particle of the 26S proteasome, played an important role in intracellular protein degradation [75]. The 20S proteasome beta 5 subunit (spot 131) was identified in this work, which could be activated and induced by drought even oxidative stress. Thus it could remove damaged proteins to a greater extent under drought stress [76]. Pathogenesis-related proteins (PRs) are defined as plant proteins induced by pathology or related circumstances [77]. In addition to protecting plants from pathogens as an essential component of plant defense responses [78], protein PR-4 can also participate in abiotic stress in plants. The expression level of *PR-4* gene in rice was strongly induced by abiotic treatments including drought, salt, and hot shock [79]. Protein PR-4 in wild wheat showed a significant upregulation in response to cold stress [80]. In wheat, wPR4e is the PR-4 family member, and can be transcriptionally activated by *F. culmorum* infection and SAR inducer treatments to respond to biotic and abiotic stresses [81]. In the 7XL/7DS translocation line, PR-4 protein (spot 128) was upregulated under drought stress (Fig. 2, Fig. 5), indicating its potential roles in plant adverse defense.

The expression of the gene under stress conditions is related to the specific interaction of the transcription factor with *cis*-elements located upstream of the regulatory region of the gene. Multistimuli responsive genes were positively correlated with the density of *cis*-elements in upstream regions [82]. In the current study, we found abundant *cis*-elements in the promoter region of 18 upregulated drought-responsive protein genes in the 7XL/7DS translocation line (Table 3). In addition to those *cis*-elements essential to growth and promoter-associated, high enrichment of *cis*-elements are relevant to MeJA-responsiveness (TGACG-motifs and CGTCA-motifs), ABRE (abscisic acid) responsiveness, MBS (drought-inducibility), defense-stress responsiveness (TC-rich repeats) and anaerobic induction (GC-motif and ARE). MeJA was an important stress-signaling observed motif that could respond to environmental stress, and thus activate plant defense mechanisms such as drought, low temperature and salt stress [83]. The *cis-acting* elements associated with anoxia condition were associated with glycolytic and fermentative gene expression, as well as in root tips and during anoxic germination of crop [38, 84]. The regulation of genes encoding enzymes in glycolysis and fermentation metabolism may be related to abscisic acid and auxin in rice to maintain sugar and ATP levels for prolonging survival [85].

To adapt to the unfavorable environment, transcription factors in plants, on the one hand, can combine with *cis*-elements located in the promoter regions of various stress response genes to activate cascades and even entire gene networks, thereby enhancing tolerance to diverse stresses at once [86]. On the other hand, physiologically, it was manifested as an excessive accumulation of ROS, which also included the increase of antioxidant enzymes [87], the degradation of protein [88] and the production of ABA [89]. The gene expression patterns of the upregulated proteins (Fig. 7) showed that they could respond to various abiotic, indicating that the corresponding *cis-acting* elements played a role in various stress environments. Thus, when subject to drought stress, some defensive protein genes were activated such as peroxidase 1 (spot 65 and spot 135), DHAR (spot 65), and 26S proteasome (spot 144 and spot 131). The synergistic expression of these proteins could form a network to enhance drought tolerance in plants.

## Conclusions

Wheat-*Th. intermedium* 7XL/7DS translocation caused better drought tolerance and significant grain proteome changes. 2D-DIGE based proteomic analysis identified 55 upregulated drought-responsive DAP spots representing 48 unique proteins caused by 7XL/7DS translocation. These proteins were mainly involved in stress defense and storage protein synthesis. In particular, the significantly upregulated proteins peroxidase 1 and 2-Cys peroxiredoxin BAS1 had a close interaction with other stress-defensive proteins. Further *cis-acting* element analysis found that the promoter regions of these upregulated DAP genes contained abundant stress responsive *cis-acting* elements such as ABRE elements and MBS, which could play important roles in response to adverse stressors. Transcriptional expression analyses by RNA-seq and RT-qPCR revealed that some drought-responsive DAP genes also highly expressed under drought stress. The synergistic response of these stress-defensive DAPs could contribute to the better drought tolerance of 7XL/7DS translocation line YW642. The drought-responsive proteins identified in Wheat-*Th. intermedium* 7XL/7DS translocation line provide potential gene resources for improving drought tolerance of wheat cultivars.

## Methods

### Wheat materials and drought treatments

The materials used in this work included common wheat cultivar Zhongmai 8601 and Zhongmai 8601-*Thinopyrum intermedium* 7XL/7DS translocation line YW642 developed by Xin et al. [36], which was collected from Institute of Crop Science, Chinese Academy of Agricultural Sciences.

Wheat materials were planted in the greenhouse of Chinese Academy of Agricultural Sciences. Two different treatments were applied during wheat growth and development: well-watered control group and water-deficit treatment group. After heading, the two groups were treated differently. The control group was watered every 3 days with 800 mL water each time, while the drought-treated group was watered every 10 days with the same amount of water. The experiment included three biological replicates, and each replicate had 20 pots and each pot contained 6-8 plants. The plants were labeled with tapes in different colors after flowering. The grain samples and mature plants from five developmental stages (15, 20, 25, 30 and 45 days post anthesis, DPA) and three biological replicates were collected and then stored at − 80 °C for later analysis.

### Measurement of main agronomic traits

After the plants were mature, 15 plants were selected from each replicate in each group to measure the main agronomic traits, including plant height, ear length, number of tillers, number of effective spikelets, number of spikelets, grain number per spike and 1000-grain weight.

### Grain protein extraction and quantitation

Proteins from wheat developing grains were extracted based on the method of Wang et al. [29] with minor modifications. Grain samples of 0.5 g were quickly ground into fine powder in liquid nitrogen for about 20 min, and then 4 mL frozen extraction buffer was added, in which 50 mL solution contained 15 g sucrose, 0.605 g Tris, 0.93 g EDTA-Na2 (ethylenediaminetetraacetic acid disodium salt), 1 g SDS (sodium dodecyl sulfate), 0.5 g PVPP (polyvinylpolypyrrolidone), 0.155 g DTT (dithiothreitol), and 0.0174 g PMSF (phenylmethylsulfonyl fluoride). An equal volume of Tris-balanced phenol was added to the sample and then ground to a homogeneous state. After centrifuging for 10 minutes at 4 °C and 13,000 rpm, the supernatant was transferred to a new 50 mL centrifuge tube. Subsequently, four volumes of 100 mM of ammonium acetate-methanol solution (0.778 g ammonium acetate, 0.31 g DTT, volumetricing to 100 mL with methanol), were added and stored in − 20 °C freezer for 12 h. After centrifuging for 30 min at 4 °C and 13,000 rpm, the supernatant was discarded, the pellet was washed three times with four volumes of pre-chilled acetone containing 0.02 g DTT/10 mL, and then centrifuged for 30 min at 4 °C and 13,000 rpm. Finally, the extracted proteins were dried under vacuum, and then dissolved with lysis buffer containing 7 M urea, 2 M thiourea, 4% 3-[(3-Cholamidopropyl)-dimethyl- ammonio]-1-propane (CHAPS) and 20 mM DTT. The concentration of the extracted grain proteins was determined by using the 2D Quant Kit (GE Healthcare, USA) with bovine serum albumin (BSA, 2 mg/mL) as the standard. The prepared protein solution was stored at − 80 °C for later use.

### Protein labeling, 2D-DIGE, 2-DE and image analysis

The differentially accumulated proteins (DAPs) during grain development in Zhongmai 8601 (CK, control) and YW642 under drought stress were identified by 2D-DIGE, and the experimental design for 2D-DIGE analysis was according to Cao et al. [90] with minor modifications. Protein samples were separately labeled with CyDyes™ (GE Healthcare, USA). Equal amounts (50 mg) of samples per gel were labeled with 400 pmol freshly dissolved Cy3 and Cy5 and the sample mixture labeled with Cy2 was used as the internal standard. After the first-dimension isoelectric focusing (IEF), the second sodium dodecyl sulfate-polyacrylamide gel electrophoresis (SDS-PAGE) was performed using 12% gel. The images were visualized using Typhoon™ 9400 scanner with filters for the excitation/emission wavelengths of each dye: Cy2 (Blue, 520 nm), Cy3 (Green, 580 nm), and Cy5 (Red, 670 nm), and then analyzed with the DeCyder software v.6.05 (GE Healthcare, USA).

Protein samples (600 μg) were further separated by conventional two-dimensional electrophoresis (2-DE) gels. The nonlinear 2-DE isolation with higher resolution was performed based on Jiang et al. [91]. After electrophoresis, the protein spot images were visualized by Coomassie Brilliant blue G-250 (Sigma, USA) staining and analyzed by ImageMaster™ 2-D platinum software version 7.0 (GE Healthcare, USA). Only those with significant and reproducible changes (abundance variation at least 1.5-fold, Student’s t-test *p* < 0.05) were regarded as DAP spots.

### Trypsin digestion and MALDI-TOF/TOF-MS

The DAP spots from 2-DE gels were excised and digested with trypsin in 2 mL centrifuge tubes as described by Lv et al. [92] After bleaching and drying, the tryptic peptides analysis was performed by ABI 4800 Proteomic Analyzer matrix-assisted laser desorption/ionization time-of-flight/time-of-flight mass spectrometry (MALDI-TOF/TOF-MS) instrument (Applied Biosystems/MDS Sciex, USA). The MS/MS spectra were searched against the NCBI wheat protein database (136,744 entries)-the nonredundant Ensembl plants database of 2021 using software MASCOT version 2.1 (Matrix Science), and the related parameter settings were according to Cao et al. [90].

### Function classification and subcellular localization

Protein function classification was according to the annotation from AgBase version 2.00. Subcellular localization of the DAPs was predicted according to the integration of WoLF PSORT (https://www.genscript.com/wolf-psort.html), UniprotKB (http://www.uniprot.org/) and Plant-mPLoc (http://www.csbio.sjtu.edu.cn/bioinf/ plant-multi/).

The transformation in Arabidopsis protoplasts of the representative drought stress-related proteins was used to further verify the results of subcellular localization prediction. pSAT1-GFP-N (Pe3449) vector was used to construct different recombinant plasmids, and then transformation in Arabidopsis mesophyll protoplasts was performed according to the previous report [93]. The GFP fluorescence signals were observed by using confocal laser scanning microscope (Leica TCS SP5, Wetzlar, Germany).

### Expression profiling and hierarchical clustering analysis of the DAPs

TBtools software v1.0971 was used to perform the expression profiling and hierarchical clustering analysis of the DAPs in spot intensity changes via heatmap analysis based on Chen et al. [94].

### Protein-protein interaction analysis

Through the Search Tool for the Retrieval of Interacting Genes/Proteins (STRING) database (version 11.5, https://string-db.org), the sequences of all identified proteins were used to construct the protein-protein interaction (PPI) network, then the results were exported and saved as a *tsv* format file [95]. Cytoscape version 3.8.2 software (https://cytoscape.org/index.html) was used to display the PPI network.

### Identification of the *cis-acting* elements

The 1500 bp of the upstream promoter regions of the key DAP genes were used to perform the *cis-acting* elements analysis. Each gene was download from Gramene website (http://www.gramene.org/), and then analyzed by PlantCARE (http://bioinformatics.psb.ugent.be/webtools/plantcare/html/.). The varied types of the *cis-acting* elements involved in abiotic stress and their respective numbers were obtained.

### RNA-seq expression analysis

The publicly accessible bread wheat (var. Chinese Spring) RNA-seq database (version RefSeq1.1) was used to perform RNA-seq expression analysis. The RNA-seq data of the genes corresponding to the regulated DAP spots under drought stress were obtained from expVIP (http://www.wheat-expression.com/) [96]. The downloaded data was tpm reads and the Log2 option was checked. The expression profiling and hierarchical clustering analysis were performed by TBtools software v1.0971.

### mRNA extraction, reverse transcription and RT-qPCR

The grain samples were ground into powder in liquid nitrogen and total RNA was isolated by using TRIzol reagent (Invitrogen, Carlsbad, CA, USA). The reverse transcription reactions were performed by using a PrimeScript RT Reagent Kit with gDNA Eraser (TaKaRa, Shiga, Japan) according to the manufacturer’s instructions. Gene-specific primers were designed by using online Primer3Plus (http://www.bioinformatics.nl/cgi-bin/primer3plus/primer3plus.cgi). Through the melting curve analysis of reverse transcription polymerase chain reaction (RT-PCR) products, the specificity of the corresponding bands in the agarose gel was identified. *Ubiquitin* was used as the reference gene. Real-time quantitative polymerase chain reaction (RT-qPCR) was performed in CFX96 Real Time system (Bio-Rad Laboratories) according to Livak and Schmittgen [97]. Three biological replicates were performed for each sample.

## Supplementary Information

**Additional file 1.**

**Additional file 2.**

## Data Availability

The data sets supporting the conclusions of this article are included within the article and its additional files. The data sets used and/or analyzed during the current study are available from the author on reasonable request (Yueming Yan: yanym@cnu.edu.cn). The mass spectrometry proteomics data have been deposited to the ProteomeXchange Consortium (http://proteomecentral.proteomexchange.org) via the iProX partner repository [98] with the dataset identifier PXD030025. All the links of datasets used in the study: WoLF PSORT (https://www.genscript.com/wolf-psort.html). UniprotKB (http://www.uniprot.org/). Plant-mPLoc (http://www.csbio.sjtu.edu.cn/bioinf/ plant-multi/). STRING (https://string-db.org). Cytoscape (https://cytoscape.org/index.html). Gramene (http://www.gramene.org/). PlantCARE (http://bioinformatics.psb.ugent.be/webtools/plantcare/html/). expVIP (http://www.wheat-expression.com/). Primer3Plus (http://www.bioinformatics.nl/cgi-bin/primer3plus/primer3plus.cgi). ProteomeXchange Consortium (http://proteomecentral.proteomexchange.org).

## Abbreviations

2D-DIGE: two-dimensional difference gel electrophoresis
DAP: differential accumulation protein
ABA: abscisic acid
ROS: reactive oxygen species
NADPH: nicotinamide adenine dinucleotide phosphate
BYDV: barley yellow dwarf virus
GISH: genome in situ hybridation
BSA: bovine serum albumin
IEF: isoelectric focusing
SDS-PAGE: sodium dodecyl sulfate-polyacrylamide gel electrophoresis
PPI: protein-protein interaction
RT-PCR: reverse transcription polymerase chain reaction
RT-qPCR: Real-time quantitative polymerase chain reaction
TCTP: translationally controlled tumor protein
TSA: 2-Cys peroxiredoxin BAS1
ALT: alanine aminotransferase 2
FTSH2: ATP-dependent zinc metalloproteinase
AGPL: ADP-glucose pyrophos-phorylase large subunit
rbcS: ribulose-1,5-bisphosphate carboxylase/oxygenase small subunit
BADH: betaine-aldehyde dehydrogenase
DHAR: dehydroascorbate reductase
atp2: ATP synthase beta subunit
BDAI: dimeric alpha-amylase inhibitor
APX: ascorbate peroxidase
UPS: ubiquitin-proteasome system
PRs: pathogenesis-related proteins
