# Supplementary material for: 2D-DIGE based proteome analysis of wheat-Thinopyrum intermedium 7XL/7DS translocation line under drought stress
Source: BMC Genomics. 2022 May 14;23:369. doi: 10.1186/s12864-022-08599-1 (PMC9107758; doi:10.1186/s12864-022-08599-1)
Supplement: Supplementary file 2 — Additional file 2. [file 12864_2022_8599_MOESM2_ESM.docx]

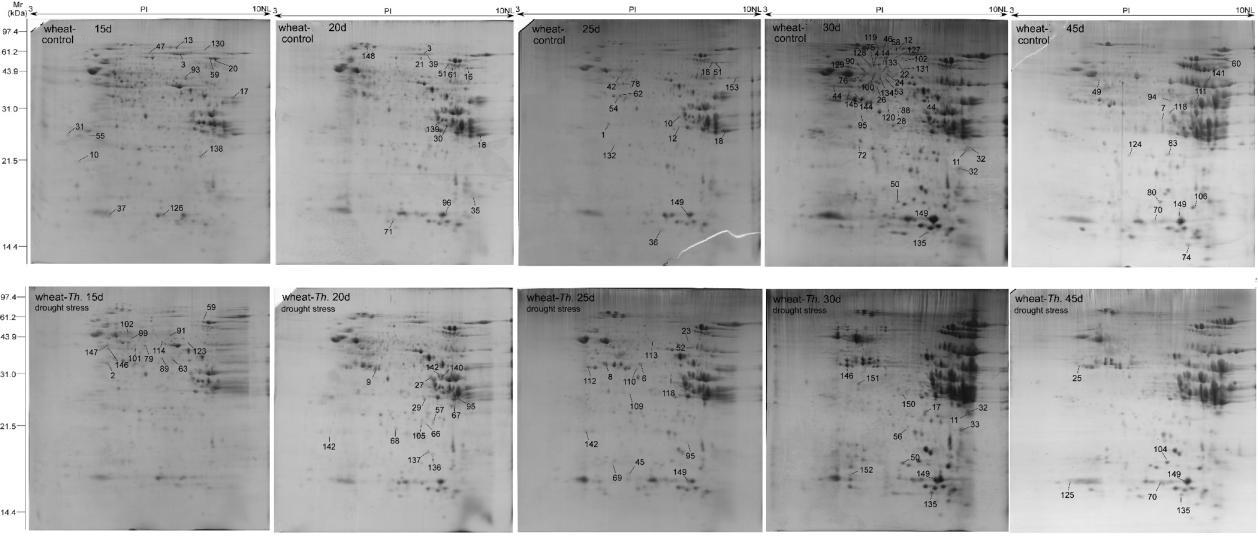


Fig. S1. Dynamic proteomic expression profiling of five grain development periods at 15, 20, 25, 30 and 45 DPA by 2-DE.

The first row in the picture is the Zhongmai 8601 under drought stress and the second row is drought stress of wheat*-Thinopyrum intermedium* 7XL/7DS translocation line.
